# Production of antibody against elephant endotheliotropic herpesvirus (EEHV) unveils tissue tropisms and routes of viral transmission in EEHV-infected Asian elephants

**DOI:** 10.1038/s41598-018-22968-5

**Published:** 2018-03-16

**Authors:** Varankpicha Kochagul, Saralee Srivorakul, Kittikorn Boonsri, Chalermchart Somgird, Nattawooti Sthitmatee, Chatchote Thitaram, Kidsadagon Pringproa

**Affiliations:** 1 Veterinary Diagnostic Laboratory, Faculty of Veterinary Medicine, Chiang Mai University, Chiang Mai, Thailand; 2 Center of Excellence in Elephant and Wildlife Research, Chiang Mai University, Chiang Mai, Thailand; 3 Department of Companion Animal and Wildlife Clinic, Faculty of Veterinary Medicine, Chiang Mai University, Chiang Mai, Thailand; 4 Department of Veterinary Biosciences and Veterinary Public Health, Faculty of Veterinary Medicine, Chiang Mai University, Chiang Mai, Thailand

## Abstract

Elephant endotheliotropic herpesvirus (EEHV) is one of the most devastating viral infectious diseases in elephants worldwide. To date, it remains unclear how elephants get infected by the virus, where the virus persists, and what mechanisms drive the pathogenesis of the disease. The present study was aimed to develop an antibody against glycoprotein B (gB) of EEHV, investigate the EEHV tissue tropisms, and provide the possible routes of EEHV transmission in Asian elephants. Samples from elephant organs that had died from EEHV1A and EEHV4 infections, peripheral blood mononuclear cells (PBMC) from EEHV4- and non-EEHV-infected calves were used in this study. The results of western immunoblotting indicated that the antibody can be used for detection of gB antigens in both EEHV1A- and EEHV4-infected samples. Immunohistochemical detection indicated that the EEHV gB antigens were distributed mainly in the epithelial cells of the salivary glands, stomach and intestines. Immunofluorescence test of PBMC for EEHV gB in the EEHV4-infected calf indicated that the virus was observed predominantly in the mononuclear phagocytic cells. The findings in the present study unveil tissue tropisms in the EEHV1A- and EEHV4-infected calves and point out that saliva and intestinal content are likely sources for virus transmission in EEHV-infected Asian elephants.

## Introduction

Elephant endotheliotropic herpesvirus (EEHV) is responsible for one of the most devastating viral infectious diseases in elephants worldwide, especially young Asian elephants (*Elephas maximus*)^1–3^. EEHV is classified in the family *Herpesviridae*, subfamily *Betaherpesvirinae*, genus *Proboscivirus*, and species *Elephantid betaherpesvirus* (https://talk.ictvonline.org/taxonomy/). Eight genotypes of EEHV have been identified thus far, including EEHV1A, EEHV1B, and EEHV2–7^1,4^. EEHV1A, EEHV1B, EEHV4, and EEHV5 are associated with, and often cause, severe hemorrhagic disease in Asian elephants, whereas EEHV2, EEHV3, EEHV6 and EEHV7 have been found in African elephants (*Loxodonta africana*) and are generally known to cause non-fatal diseases^1,3,5^. EEHV1A and EEHV1B are by far the most common types with high fatality rates in young Asian elephants of age 1 year to 4 years^1,3,6^. Reports of EEHV-infected Asian elephants demonstrate that most of the EEHV-infected calves develop the clinical signs of lethargy, anorexia, lameness, colic, and diarrhea, with, often, many of them subsequently dying within an hour to 7 days after showing the clinical signs^3,5^. So far, there are no vaccines and no specific treatment for the disease. Treatment of EEHV-infected calves with antiviral drugs, such as famciclovir and acyclovir, has been shown to be a success in some cases, indicating that the results of antiviral drug therapy are uncertain^7–9^.

Presently, pathogenesis of EEHV in elephants remains unknown and data demonstrating EEHV pathogenesis in elephants are extremely scant. This might be due to the fact that propagation of EEHV in the cell culture system has not been successful thus far^1,2,10^. Moreover, limitations with regard to laboratory confirmation of EEHV-suspected cases in some areas may narrow down information about EEHV pathogenetic study. Diagnosis and confirmation of EEHV are largely based on pathological findings, followed by detection of the viral nucleic acid^2,5,6,11^. The significant gross and histopathological findings observed in EEHV-infected cases are hemorrhages of the internal organs and usually the presence of intranuclear inclusion bodies, predominantly in the endothelial cells^2,3,5^. A previous study of EEHV genome distribution revealed that the viral genome was shown to have been observed in the heart, followed by in the tongue and the liver, in that order^10^. However, it is unclear whether the viral genome was found within the blood vessels or the tissue parenchyma since DNA extraction was done on the whole tissue lysates. On the other hand, several questions have been raised and remain unknown, such as what mechanisms the virus uses for disease transmission and dissemination through the body, what the target organs of the virus are, and where the virus persists during the chronic phase of virus infection. Although infectious virus particles have been detected in the saliva or trunk washes of EEHV-infected elephants^11–13^, it remains to be determined whether saliva serves as a source for viral transmission, and if so, what the anatomical sites are for viral replication and egress of the virus in the saliva.

Pathogenetic studies of viral infectious diseases have long been largely documented by the use of the antigen-antibody detection system^14–17^. The advantage of using antigen-antibody detection to explain the disease pathogenesis is that one may visualize tissue tropism of virus-infected cells directly by using microscopes or other sophisticated tools^14–17^. In EEHV, however, there are no commercial antibodies available, to date. Moreover, to the best of the author’s knowledge, although an antibody against EEHV has been generated^18^, the use of such an antibody to evaluate EEHV antigen distribution in EEHV-infected cases has not been addressed thus far. In the present study, polyclonal antibodies against glycoprotein B (gB) of EEHV were produced and validated. Thereafter, distribution of EEHV1A and EEHV4 gB antigens in elephant samples was investigated by immunohistochemistry and immunofluorescence. Furthermore, this study demonstrated tissue tropisms and provided evidence for the possible routes of viral transmission in EEHV1A- and EEHV4-infected Asian elephants.

## Results

### Gross lesions and molecular analyses of EEHV infection in elephant calves

**Figure 1:**
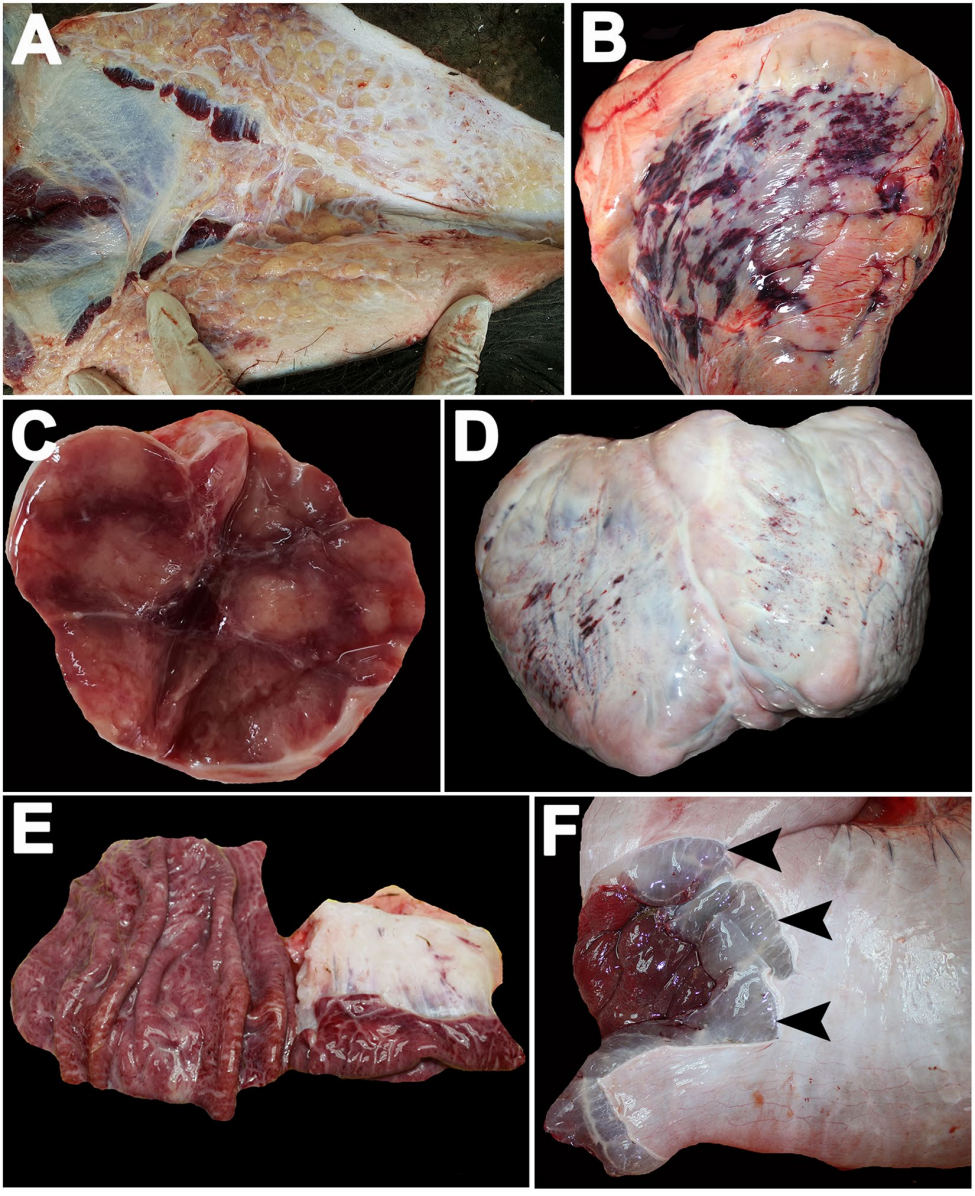
Gross findings of elephant carcasses case 1 and case 4. Representative images of calves infected by EEHV1A (**A–C**) and EEHV4 (**D–F**). Infection of EEHV1A in the calves revealed petechial and ecchymotic hemorrhages with edema throughout the internal organs, including the subcutaneous tissue of the sublingual area (**A**), heart (**B**), and lymph node (**C**). EEHV4 infection in the calf resulted in mild hemorrhages of the heart (**D**), but caused severe hemorrhages and edema (arrowhead) in the submucosal and the subseroal areas of the stomach (**E**) and rectum (**F**).

At necropsy, elephant carcasses cases 1–4 revealed petechial and ecchymotic hemorrhages with edema throughout the internal organs, including the subcutaneous tissues, hearts, lymph nodes, and intestines (Fig. 1). Molecular analyses by conventional polymerase chain reaction (PCR) and subsequently gene sequencing for polymerase and terminase genes of EEHV confirmed the EEHV1A infection in case 1 and case 2, while in case 3 and case 4, it was EEHV4 infection. It should be noted that hemorrhages of the hearts were shown to be severe in the EEHV1A-infected calves, while in the EEHV4-infected calves, less severity was observed (Fig. 1B,D). However, hemorrhages and edema of the submucosal and the subserosal areas of the stomachs and intestines have been predominantly noticed in elephants infected by EEHV4 (Fig. 1E,F). Case 5 reported negative for EEHV infection by PCR test of both polymerase and terminase genes. Furthermore, since elephant gamma herpes virus (EGHV) has been shown to be frequently observed in elephants with no obvious clinical significance, the presence of EGHV in the studied samples was then tested by PCR. The results showed that all the animals were negative with regard to EGHV infection (data not shown).

### Production and characterization of polyclonal antibodies against EEHV

Since envelop gB is one of the most highly conserved regions in the herpesviruses^19–22^ and the presence of herpesvirus gB in a specific cell type might indicate the target cells of herpesviruses, polyclonal antibodies against EEHV gB were developed. Peptide sequence (EPSTKFKVYKDYERLQ) that belongs to the amino acid positions 259–274 of EEHV gB from accession no. AF411189 was selected and synthesized. After five injections of peptide-conjugated keyhole limpet hemocyanin (KLH) carrier protein had been made to the New Zealand White rabbits, the sera containing polyclonal anti-EEHV gB antibodies were collected and characterized. The sodium dodecyl sulfate polyacrylamide gel electrophoresis (SDS-PAGE) followed by Coomassie Blue stain revealed the expected size of immunoglobulin protein to be ~25 kDa and ~50 kDa, which indicated the light and the heavy chains of the immunoglobulin G (IgG), respectively (Fig. S1). It should be noted that no difference was observed in the signal intensity of the rabbit sera immunized with different peptide concentrations.

### Detection of EEHV1A and EEHV4 gB in elephant tissues by western immunoblotting

**Figure 2:**
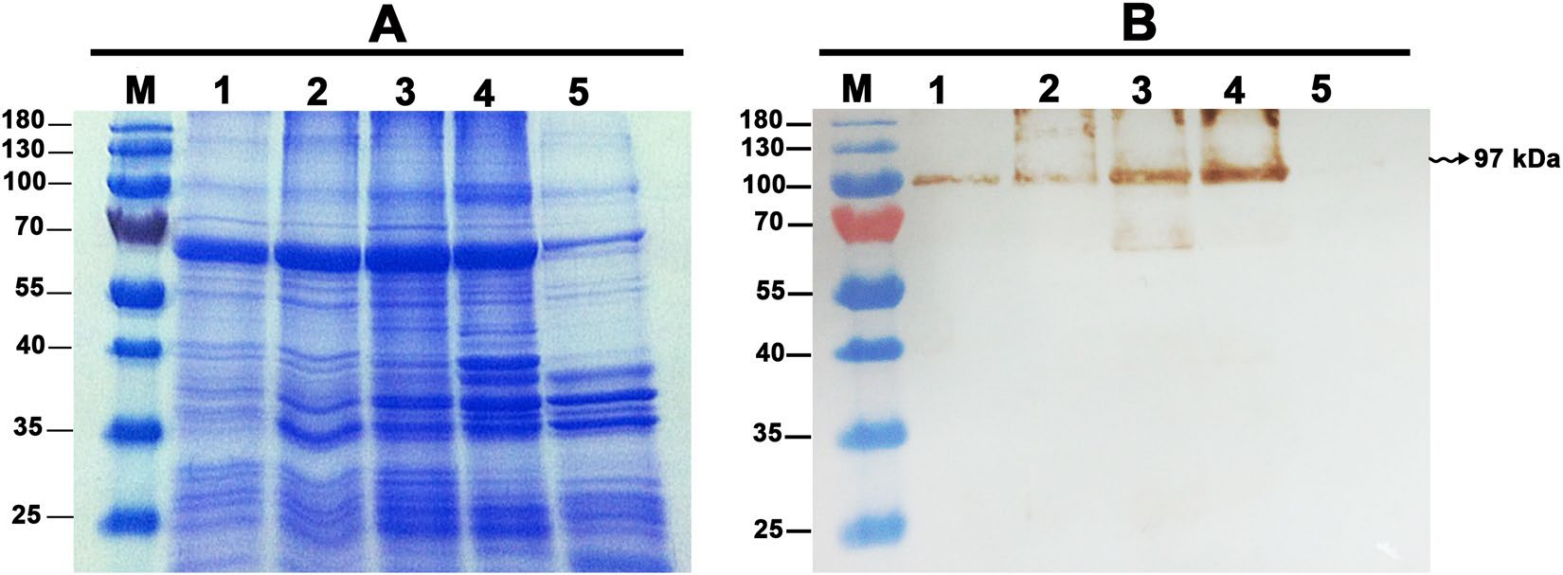
SDS-PAGE of tissue lysates from EEHV1A-infected, EEHV4-infected, and non-EEHV-infected calves. SDS-PAGE gel of the elephant tissue lysates stained with Coomassie Blue (**A**) and immunostained with polyclonal anti-EEHV gB antibodies (**B**). The anti-EEHV gB antibodies demonstrated a positive signal against EEHV1A gB (lane 1 and lane 2) and EEHV4 gB (lane 3 and lane 4) at molecular weights of ~97 kDa and ~98 kDa, respectively, compared to the protein molecular weight marker (lane M), while they showed a negative signal in the elephant sample that died from non-EEHV-infection (lane 5). Lane M = protein molecular weight marker; lane 1 = total protein from case 1; lane 2 = total protein from case 2; lane 3 = total protein from case 3; lane 4 = total protein from case 4; and lane 5 = total protein from case 5.

SDS-PAGE followed by Coomassie Blue stain of the whole protein lysates from the frozen tissues of EEHV1A-infected (Fig. 2A, lanes 1–2), EEHV4-infected (Fig. 2A, lanes 3–4), and non-EEHV-infected calves (Fig. 2A, lane 5) revealed that the extraction procedure could obtain the protein yield at high levels. Following this, the SDS-PAGE gel was transferred to the nitrocellulose membrane and immunostained with anti-EEHV gB antibodies. The signals of the bands that corresponded to the predicted protein sizes of approximately 97 kDa and 98 kDa of EEHV1 and EEHV4 gB, respectively, were observed (Fig. 2B). The negative control, which was the tissue lysate obtained from the elephant that died due to non-EEHV infection, showed no signal (Fig. 2B, lane 5). It has to be stressed as a fact that the gB protein detected by western blot in the present study was not from EGHV since PCR analyses revealed it as negative for gB and the polymerase genes of EGHV from the studied animals. These results confirm that the rabbit polyclonal anti-EEHV gB antibodies produced in the present study were specific to the gB of EEHV and were able to detect both EEHV1A- and EEHV4-genotypes in Asian elephants.

### Histopathology and immunohistochemical evaluation of EEHV-infected calves

**Figure 3:**
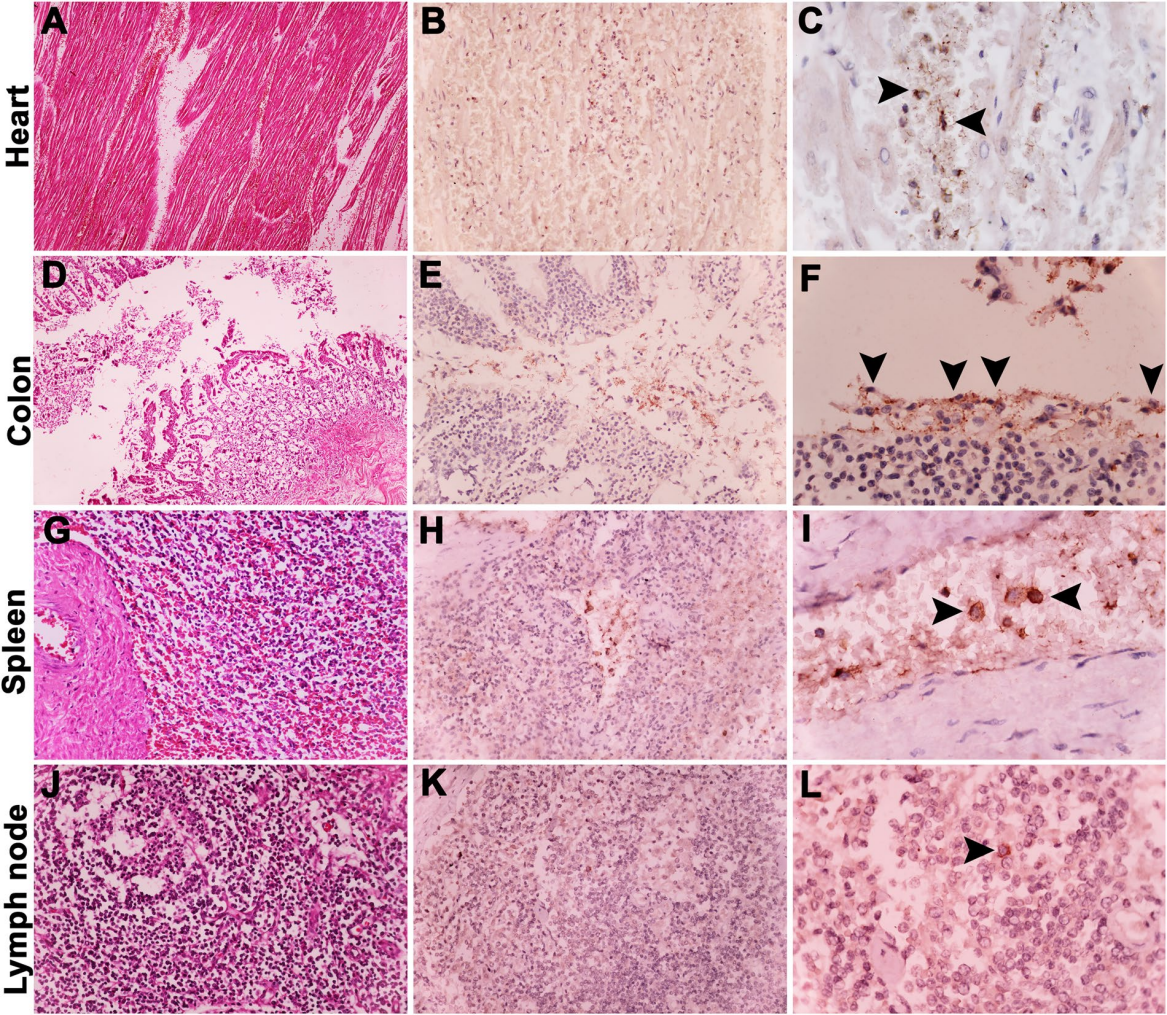
Representative images of histopathological and immunohistochemical findings of the EEHV1A-infected calf. Sections of the heart (**A–C**), colon (**D–F**), spleen (**G–I**), and lymph nodes (**J–L**) of the EEHV1A-infected calf showed mild-to-moderate degree of hemorrhages with infiltration of lymphohistiocytic inflammatory cells. Immunohistochemical labeling with anti-EEHV gB antibodies revealed positive signals in the cytoplasm of the mononuclear phagocytic cells within the blood vessels of the heart (**B,C**) and spleen (**H,I**). The colon showed positive signals (arrowhead) in the cytoplasm of intact villi (**E,F**) and necrotic cells within the intestinal lumen. The lymph nodes showed rarely immunolabeling positive cells (arrowhead) of the lymphoid follicle (**K,L**). The negative controls were sections of EEHV1A-infected calves that were incubated with normal rabbit serum instead of rabbit anti-EEHV gB antibodies, or incubation of non-EEHV-infected tissue sections with rabbit anti-EEHV gB antibodies.

Histopathological findings of EEHV1A- and EEHV4-infected calves revealed multifocal hemorrhages, vasculitis, and lymphohistiocytic inflammation of the vessels in the internal organs, such as the heart, spleen, lymph nodes, and colon (Fig. 3). The gastrointestinal tract revealed mild-to-moderate degree of hemorrhages with sloughing off of the mucosal epitheliums. The spleen and the lymph nodes had moderate lymphoid depletion and necrosis (Fig. 3G,K). Immunohistochemical labeling of EEHV gB demonstrated positive signals distributed throughout the internal organs, predominantly in the salivary glands of EEHV1A-infected cases and in the intestinal epitheliums of EEHV4-infecetd cases (Table 1). Moreover, immunolabeling positive cells were also shown to be observed in the peripheral blood mononuclear cells (PBMCs) within the blood vessels, such as the heart and spleen (Fig. 3B–C,H,I), and the lymphoid follicles of the lymph node (Fig. 3K,L). Intact villi and necrotic epithelial cells of the colon showed a positive signal for the anti-EEHV gB immunolabeling (Fig. 3D–F). The blood mononuclear cells in the internal organs, such as the spleen and the lymph nodes, were observed for EEHV gB immunolabeling (Fig. 3H,I,K,L). Tissues from case 5 were shown to be negative for EEHV gB by immunohistochemical labeling (Table 1).

**Table 1:** Detection of EEHV gB by immunohistochemistry in selected organs.

Organ	Case*				
	1	2	3	4	5
Spinal cord	−	−	n.a.	−	n.a.
Lymph nodes	+	+	+	+	−
Spleen	++	++	++	++	−
Lung	++	++	+	++	−
Liver	++	+	+	+	−
Kidney	+	+	+	+	−
Heart	++	++	++	+	−
Tongue (with salivary glands or salivary ducts)	+++	+++	+	++	−
Stomach	++	+	+	+++	n.a.
Duodenum	+	−	n.a.	+	n.a.
Jejunum	n.a.	n.a.	n.a.	+	n.a.
Ilium	−	−	+	+	−
Colon	−	−	++	+	−
Cecum	n.a.	−	++	+	n.a.
Rectum	n.a.	n.a.	n.a.	+++	n.a.

^*^Occurrence and intensity of signal: − = no signal; + =mild; ++ = moderate; +++ = high; n.a. = not available.

### Salivary glands serve as tissue tropisms for EEHV1A and EEHV4 infections

**Figure 4:**
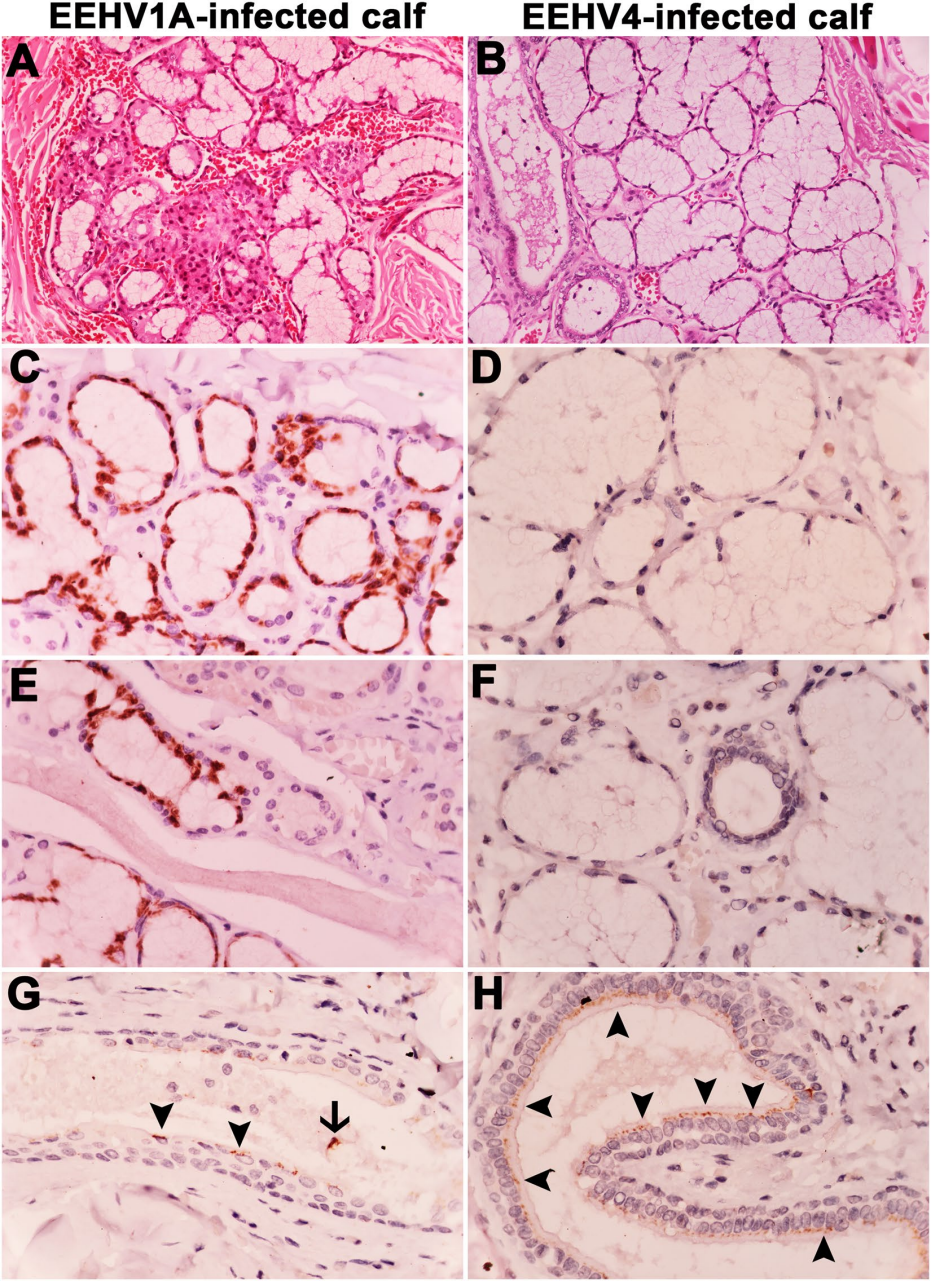
Histopathological and immunohistochemical findings of the salivary glands of EEHV1A-infected and EEHV4-infected calves. Representative images of sections from the salivary gland of the EEHV1A-infected calf (**A,C,E,G**) and the EEHV4-infected calf (**B,D,F,H**). Infection with EEHV1A revealed moderate-to-severe, multifocal, lymphohistiocytic inflammation of the serous and the mucous acini, while less severity was observed in the EEHV4-infected calf (**A,B**). Immunohistochemical labeling of EEHV gB in the EEHV1A-infected calf indicated strong positive signals in the cytoplasm of the epitheliums of serous and mucous acini (**C,E**). EEHV gB immunolabeling positive cells were also shown to be observed in the cytoplasm of the cuboidal and columnar epithelial cells of the striated and the interlobular ducts (arrowhead, **G,H**). Some EEHV gB immunolabeling positive cells (arrow) were found within the lumen of the salivary ducts (**G**). The negative controls were sections of EEHV1A- and EEHV4-infected calves that were incubated with normal rabbit serum instead of rabbit anti-EEHV gB antibodies, or incubation of non-EEHV-infected tissue sections with rabbit anti-EEHV gB antibodies.

**Figure 5:**
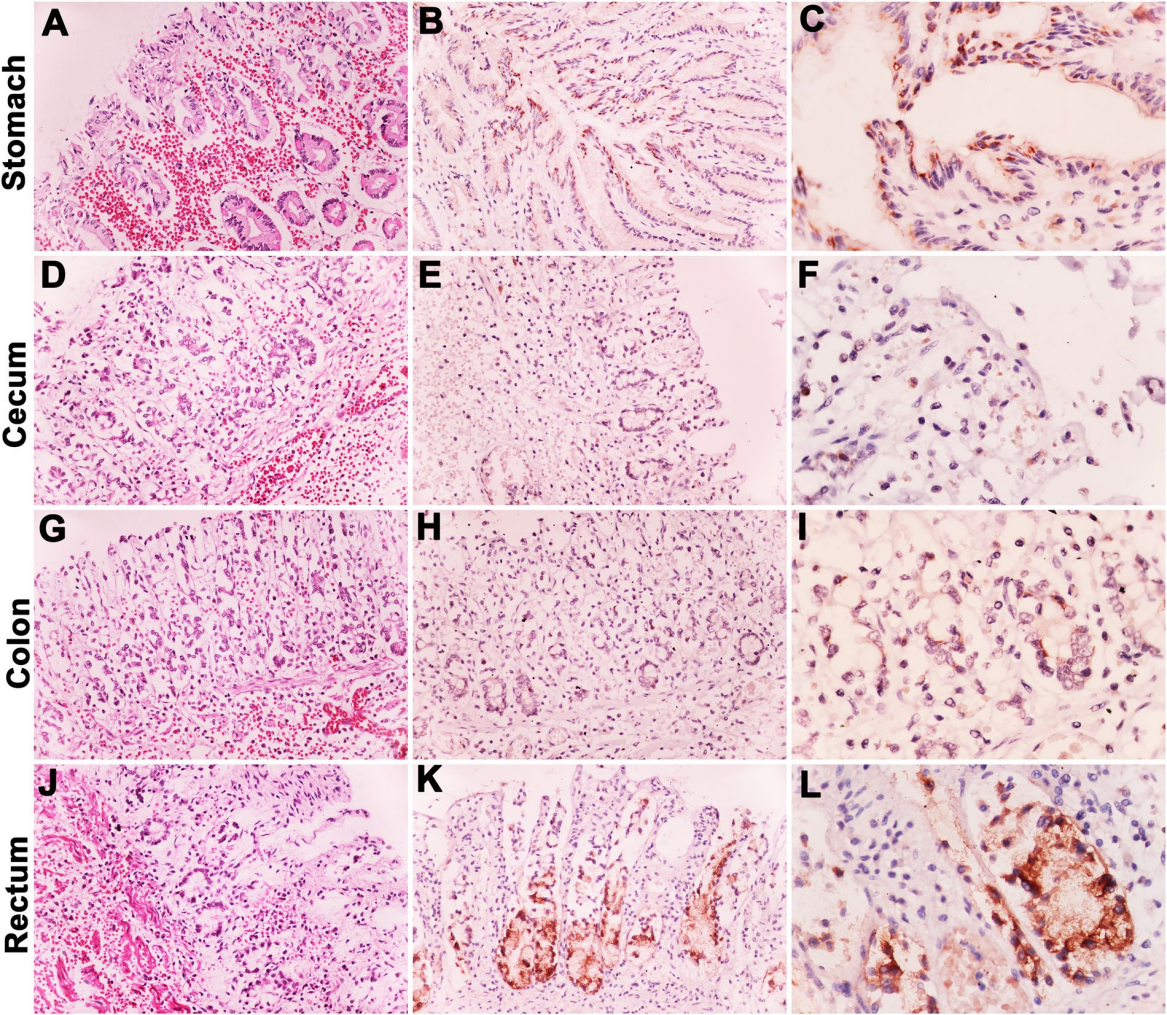
Representative images of histopathological and immunohistochemical findings of the EEHV4-infected calf. Sections of the stomach (**A–C**), cecum (**D–F**), colon (**G–I**), and rectum (**J–L**) of the EEHV4-infected calf showed severe hemorrhage and edema of the laminar propria and submucosa layer, with moderate degree of lymphohistiocytic cell infiltration. Immunohistochemical labeling with rabbit anti-EEHV gB revealed strong and diffused positive signals in the cytoplasm of the gastric mucosa (**B,C**), while less positive cells were observed in the cecum (**E,F**) and colon (**H,I**). The strong intensity of the positive signals in the basal and/or crypts of the intestinal epithelium of the rectum were noticeable (**K,L**). The negative controls were sections of EEHV4-infected calves that were incubated with normal rabbit serum instead of rabbit anti-EEHV gB antibodies, or incubation of non-EEHV-infected tissue sections with rabbit anti-EEHV gB antibodies.

Sublingual salivary glands of the EEHV1A-infected calves (case 1 and case 2) showed cellular and nuclear enlargement, with moderate lymphohistiocytic inflammatory cell infiltration (Fig. 4A), while less severity was observed in the sublingual salivary glands of the EEHV4-infected calf (case 4) (Fig. 4B). Immunohistochemical labeling for EEHV gB revealed that the positive signals were predominantly observed in the epitheliums of the mucous and serous acinar cells of the salivary glands of the EEHV1A-infected cases (Fig. 4C,E), while low signal was observed in the EEHV4-infected calf (Fig. 4D,F). Moreover, signals of the gB immunolabeling positive cells were also observed in the interlobular ducts and the striated ducts of the salivary glands from both EEHV1A- and EEHV4-infected calves (Fig. 5G,H). Within the ducts, the viral antigens seem to be localized in the intact ductal cuboidal and columnar cells (arrowhead) and necrotic epithelial cells in the lumen (arrow).

### Gastrointestinal system serves as tissue tropism for EEHV1A and EEHV4 infections

Histopathological findings of the gastrointestinal system in the EEHV1A- and EEHV4-infected calves revealed severe hemorrhage of the gastric and the intestinal mucosa, especially in the EEHV4-infected calf (Fig. 5). Epithelial cell necrosis and villous atrophy were presented throughout the small and the large intestines and accompanied by infiltration of the inflammatory cells. The inflammatory cells were mainly lymphocytes, macrophages, and plasma cells. Immunohistochemical labeling of EEHV gB in EEHV1A- and EEHV4-infected calves were observed mainly in the stomach and the large intestine, especially crypt epitheliums of the rectum of EEHV4-infected calf (Fig. 5K,L).

### Immunofluorescence of non-EEHV-infected and EEHV4-infected elephant PBMCs

**Figure 6:**
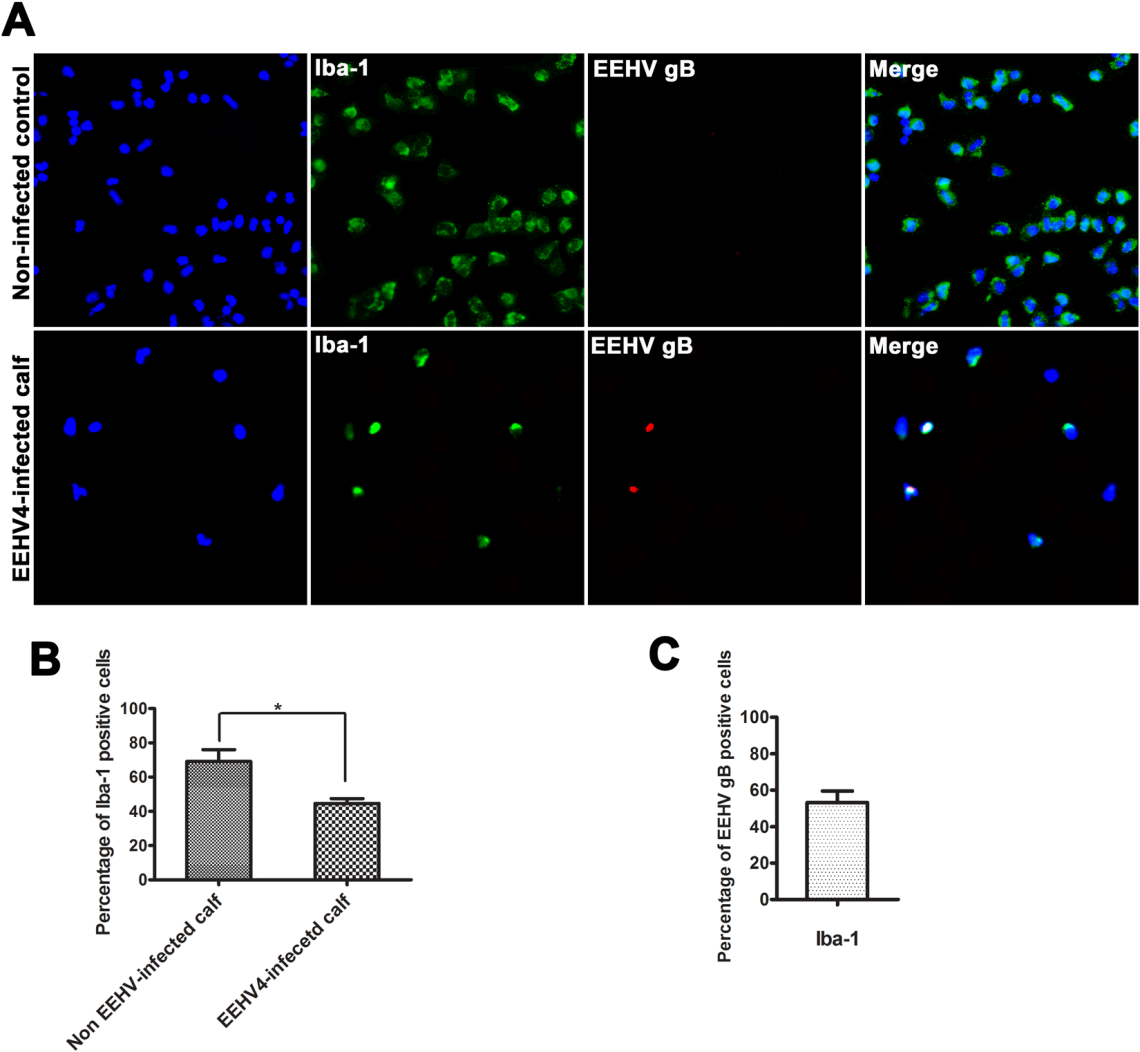
Double immunofluorescence test and quantitative assessment of PBMCs from non-EEHV-infected and EEHV4-infected calves. PBMCs obtained from healthy, non-EEHV-infected calf and fresh carcass of an elephant that had died from EEHV4 infection were immunolabeled with mouse anti-Iba-1 (green in **A**) and rabbit anti-EEHV gB antibodies (red in **A**). The nuclei were detected using bisbensimide fluorescence stain (blue in **A**). The percentage of Iba-1 immunolabeling positive cells of PBMCs in the EEHV4-infected calf was significantly lower than that in the non-EEHV-infected calf (**B**). More than 50% of the Iba-1-positive cells were immunolabeling positive for anti-EEHV gB antibodies (**C**). Negative controls were PBMCs from non-EEHV-infected calf incubated with the anti-EEHV gB antibodies, or PBMCs from the EEHV4-infected calf incubated with normal rabbit serum. Asterisks indicate statistically significant difference between EEHV4-infected and non-EEHV-infected calves (*p* < 0.05).

Immuno-fluorescent staining of PBMCs from the non-EEHV-infected calf revealed that more than 70% of PBMCs from healthy elephant were positive for Iba-1 immunolabeling (Fig. 6A). However, the Iba-1 immunolabeled positive cells were significantly less in the EEHV4-infected calf compared to the non-EEHV-infected calf (Fig. 6B). Double immunostaining of PBMCs from EEHV4-infected calf with anti-Iba-1 and anti-EEHV gB antibodies indicated that more than 50% of the Iba-1-positive cells were also positive for anti-EEHV gB immunolabeling (Fig. 6C).

## Discussion

In this study, polyclonal antibodies against EEHV gB were generated and their specificity against EEHV1A-infected and EEHV4-infected elephant calf samples was validated using various detection methods. In addition, hypothesis underlyings the pathogenesis of EEHV infection in Asian elephants was proposed. Envelop gB is one of the most highly conserved glycoproteins in the family *Herpesviridae*, and is believed to play a part together with glycoprotein H (gH) and glycoprotein L (gL) in membrane fusion and entry of the virus into infected-cells^19–22^. Moreover, gB is a major determining factor of virus infectivity both *in vivo* and *in vitro*^23,24^. The gB of herpes simplex and equine herpes virus has been shown to be involved in the second step of nuclear egress and de-envelopment in the maturation phase of herpesviruses^24,25^, which is believed to take place in the trans-Golgi network (TGN) in the cytoplasm of the infected cell^23,26,27^. Therefore, demonstration of herpesvirus gB in specific cell types might indicate either its attachment to the cells or its preparation for egress of progeny viruses from infected cells. In EEHV, gB is encoded by the U39 gene, which translates to a yield of protein of about 153 amino acids (~97–98 kDa), and is predicted to be the potential antigenic determinant for detection of EEHV^28^. A previous report has shown that polyclonal antibodies against EEHV gB can be used for EEHV antibody detection in Asian elephants by sandwich ELISA^18^. However, to the best of the researcher’ knowledge, an antibody against EEHV has never been addressed to demonstrate EEHV antigens in Asian elephants. The present study is among the earliest reports to describe the application of EEHV gB antibodies for antigen detection. The study showed that rabbit polyclonal anti-EEHV gB antibodies can be used for detection of EEHV1A and EEHV4 antigens by western immunoblotting, immunohistochemistry, and immunofluorescence test.

The findings of the present study indicate that salivary glands and gastrointestinal epitheliums are likely to serve as the target tissues for EEHV1A and EEHV4 infections. It is known that viruses in the family *Herpesviridae* comprise two distinct stages in their life cycle, including lytic replication and latency^27,29–31^. More specifically, viruses in the subfamily *Betaherpesvirinae*, such as cytomegalovirus and human betaherpesviruses, prefer to infect and replicate in the salivary glands as the primary infection site and usually use monocytes/macrophages for viral spreading through the target tissues in the body^29,32,33^. At the same time, they also use salivary glands and cells in the lymphoreticular system as sites of latency during persistent infection^29,32,33^. Salivary glands are a privileged site for cytomegalovirus latency because they inhibit the function of CD4+ and CD8+ T cells by up-regulating the expressions of IL-10 and programmed death 1 (PD1)-mediated signaling, respectively^34^. Nevertheless, since the present study demonstrated that salivary glands harbor EEHV gB in the EEHV1A-infected and EEHV4-infected calves, it is reasonable to hypothesize that the primary replication site for EEHV1A and EEHV4 may be in these glands and that the infectious virions are shaded in the saliva. This hypothesis could be further explained by the fact that EEHV1A viral nucleic acid has been observed in the saliva and elephant trunk washes of the acute fatal EEHV1A-infected calves^35^. It has been shown that dissemination of EEHV viral nucleic acid in blood (DNAemia) was detected several days prior to the observation of the clinical signs^7,35^, and shedding of EEHV1A in trunk washes has been observed to commence 10 days after the initial detection of DNAemia^7^. Moreover, since elephant trunk washes from healthy and asymptomatic elephants in the EEHV-infected herd were also found positive for viral nucleic acid^13^, it is suggested that tissues, such as salivary glands, may also serve as a site of latency for EEHV during persistent infection. It should be noted that elephant calves of cases 1–3 had succumbed to the acute infection and died after 1–7 days of showing the first clinical signs, while case 4 had died 1 month after the manifestation of the first clinical signs. Hence, immunocytological labeling of EEHV1A and EEHV4 gB in cases 1–3 was found mainly in the mucous and the serous acini of the salivary glands, while in case 4, it was shown to be in the epithelium of the striated and the interlobular ducts.

In contrast to the EEHV1A infection, the distribution of the gB antigen in the EEHV4-infected calf was predominantly observed in the gastrointestinal mucosa, especially the stomach and the rectum. Although one of the clinical signs observed in EEHV-infected cases has been diarrhea^3,9^, so far, there have been no reports of any tests conducted for the presence of EEHV in stool or intestinal content. Infection of EEHV4 in elephants has been shown to induce severe hemorrhages in the internal organs, including the intestine^6^. As found in case 4, despite recovering from the first episode of illness, at one month later the animal developed a second episode of the EEHV clinical signs, including hemorrhagic diarrhea. The findings in the present study provided the possibility that EEHV4, and probably EEHV1A, could also be detected in the intestinal content of EEHV-infected cases, especially those that presented hemorrhagic diarrhea. Although the EEHV1A-infected elephant calves investigated in this study did not present hemorrhagic diarrhea, the findings of EEHV gB in the gastric mucosa by immunohistochemistry indicate that EEHV1A is likely to be detected in the intestinal content. Therefore, it is logical to speculate that shedding of EEHV1A and EEHV4 could occur through oral and intestinal secretions.

This study showed that elephant PBMCs may play a role in the pathogenesis of EEHV infection in Asian elephants. Previously, PBMCs of mammals have been proposed to be “Trojan horses” that facilitate the spread of herpesviruses, such as equine herpesvirus-1 (EHV-1) and human cytomegalovirus (HCMV), to the target organs in the body^36,37^. More specifically, CD17+ monocytic cells are the main carrier cells of EHV-1 during the primary infection for dissemination of EHV-1 to target organs before secondary replication occurs in the endothelial cells lining the blood vessels of those organs^36,38,39^. As shown in the present study, Iba-1 positive monocytic cells are targeted by EEHV. Moreover, the distribution of EEHV gB were observed mainly in the PBMCs within the lumen of the blood vessels of the internal organs. By taking these pieces of information together, it can be hypothesized that the primary infection of EEHV1A and EEHV4 may be occurring in the salivary glands. Thereafter, the virus gets disseminated through the body via a cell-associated viremia in PBMCs to the target organs, such as the endothelial cells of the internal organs and the tissues in the gastrointestinal system. Thereafter, secondary replication of the virus may occur in the endothelial cells lining the blood vessels of those organs or the epithelial cells of the gastrointestinal system. As a result, hemorrhages, vasculitis, and ischemic thrombosis may lead to severe symptoms. However, it should be noted that the antibody produced in the present study can only be used to indicate the sites of viral attachment or maturation, and does not enable the indication of the site of viral latency. To identify the sites of latency of herpesvirus infection, the viral genomes and/or tissue responses within the infected cells needed to be examined. In conclusion, the findings in the present study unveil the possible routes of EEHV transmission as salivary and intestinal secretion, and point out that salivary glands and gastrointestinal epitheliums may be the replication sites of the virus in EEHV1A and EEHV4 infections. Moreover, PBMCs may function as carrier cells for transporting the virus to the target cells of the vital organs, and destroyed the endothelium of the capillaries which caused hemorrhagic disease (HD)-symptoms in Asian elephants.

## Methods

### Ethical statement

The study was conducted in accordance with the international ethical guidelines for animal experimentation, and all experiments were approved by the Institutional Animal Care and Use Committee, Faculty of Veterinary Medicine, Chiang Mai University, Chiang Mai, Thailand (permission number: FVM-ACUC.R12/2559).

### Necropsy and collection of elephant samples

Elephant calves from private elephant camps, Chiang Mai, Thailand, that had died from infection of EEHV1A (case 1 and case 2) and EEHV4 (case 3 and case 4), during 2015–2017 were investigated in the present study (Table 2). An elephant calf of age 1day that had died from unrelated EEHV infection (case 5) was used as the negative control. Necropsy of the animals was performed at the field of the private elephant camps (cases 1–3) or at the Veterinary Diagnostic Laboratory, Faculty of Veterinary Medicine, Chiang Mai University (case 4 and case 5). Tissue samples were collected and kept either at −20 °C or fixed in 10%-neutral buffered formalin. In addition, the whole blood of one of the EEHV4-infected calves (case 4) was collected subsequent to the animal’s death and PBMCs were obtained, as described below. The PBMCs of case 4 were investigated in comparison with PBMCs from a healthy, non-EEHV-infected calf (case 6).

**Table 2:** History of animals and specimens used in this study.

Animal	Sex	Age	PCR result	Specimen	Comment
Case 1 (HS)	Female	2 years	EEHV1A	Spinal cord, lymph nodes, heart, lung, liver, kidney, spleen, tongue, stomach, duodenum, ileum, colon	1 week of illness, treatment with famciclovir (15 mg/kg, P.O.)
Case 2 (KP)	Female	3 years	EEHV1A	Spinal cord, lymph nodes, heart, lung, liver, kidney, spleen, tongue, stomach, duodenum, ileum, colon, cecum	1 day of illness, treatment with acyclovir (15 mg/kg, P.O.)
Case 3 (PI)	Female	3 years	EEHV4	Lymph nodes, heart, lung, liver, kidney, spleen, tongue, stomach, ileum, colon, cecum	2 days of illness, no antiviral drug was given
Case 4 (PY)	Female	2 years	EEHV4	Whole blood, spinal cord, lymph nodes, heart, lung, liver, kidney, spleen, tongue, stomach, duodenum, jejunum, ileum, colon, cecum, rectum	Treatment with acyclovir (15 mg/kg, P.O.) for 3 weeks, recovery, then sudden onset and death at week 4 after first clinical signs were observed
Case 5 (DP)	Male	1 day	Negative	Lymph nodes, heart, lung, liver, kidney, spleen, tongue, ileum, colon	Still born
Case 6 (SY)	Male	5 years	Negative	Whole blood	Healthy, alive

P.O.: per os.

### Tissue processing and histopathology

The samples fixed in 10%-neutral buffered formalin were dehydrated and embedded in paraffin blocks. Histological staining of the formalin-fixed, paraffin-embedded (FFPE) samples was done on 4 μm-thick sections by hematoxylin and eosin (H&E) stain. Additional 4–5 sections/block were cut and obtained on the 3-aminopropyl-triethoxysilane coated-slides for further immunohistochemistry test, as described below.

### Polymerase chain reaction (PCR) and gene sequencing

The total DNA of the frozen tissues sampled from the heart, spleen, and lymph nodes of EEHV-infected calves (cases 1–4), non-EEHV-infected calf (case 5), and whole blood of non-EEHV-infected calf (case 6) was extracted and investigated using a commercial DNA extraction kit (Machery-Nagel GmbH, Dauren, Germany), as suggested by the manufacturer. The oligonucleotide primers of the polymerase and the terminase genes of EEHV were analyzed as previously described^3^. In addition, primers of polymerase and gB genes of EGHV derived from the GenBank accession no. EU085379.1 (Table S1) were also investigated. Conventional PCR was performed under the following conditions: 95 °C for 2 min, 35 cycles of 95 °C for 30 sec, 55 °C for 30 sec, and 72 °C for 1 min, and a final extension at 72 °C for 2 min. The PCR products were analyzed by electrophoresis, and specific bands were observed under a UV illuminator. The PCR products corresponding to the expected genome size were then further analyzed by DNA sequencing, as previously described^6^.

### Production of polyclonal antibodies

Production of polyclonal antibodies against the dominant epitope of EEHV gB was carried out, as previously described^18^. Briefly, the peptide sequence (EPSTKFKVYKDYERLQ) that belongs to the amino acid positions 259–274 of gB from accession no. AF411189 was selected and synthesized (GenScript, Piscataway, NJ), according to a previous study^18^. This peptide sequence was selected because it had 94% identity with the amino acid sequence of EEHV1A gB (accession no. KC618527.1) and 75% identity with the amino acid sequence of EEHV4 gB (accession no. KT832477.1). The peptide was coupled to the KLH carrier protein (Imject® EDC Carrier Protein Spin Kits, Thermo Scientific, Waltham, MA), according to the manufacturer’s instructions. The peptide-conjugated KLH carrier protein was titrated to different concentrations (125 µg/mL, 250 µg/mL and 450 µg/mL), and aliquot 500 µL/tube, and then stored at −20 °C for further use in the immunization study.

For immunization, the 500 µL of the peptide-conjugated KLH carrier protein solutions at the concentrations of 125 µg/mL, 250 µg/mL, and 450 µg/mL were mixed with an equal volume of Montanide (Seppic, Paris, France) and immunized 5 times subcutaneously, at 2 weeks’ interval, on the female of age 8 weeks New Zealand White rabbits (2 rabbits/peptide concentration). Prior to each immunization, 2 mL of blood was collected from the saphenous veins for antibody determination, and the last blood sampling was done 2 weeks after the final injection. Rabbit sera containing anti-EEHV gB antibodies were obtained by centrifugation, aliquot, and stored at −20 °C for further studies.

### Antibody characterization

Rabbit anti-EEHV gB antibodies immunized with different concentrations of peptide-conjugated KLH carrier protein were subjected to either SDS-PAGE and stained with Coomassie Blue, as previously described^40^, or characterized for their specificity by western immunoblot analysis, immunohistochemistry, or immunofluorescence test, as described below.

### Western immunoblotting

Proteins or cell lysates from the frozen tissues of the heart, lung, spleen, liver, kidney, tongue, and intestine of EEHV1A-infected (case 1 and case 2), EEHV4-infected (case 3 and case 4), and non-EEHV-infected (case 5) calves were extracted and subjected to SDS-PAGE. Briefly, 500 mg of homogenized tissue samples were incubated with lysis buffer (100 mM Tris HCL, 0.5 mM EDTA, β-mercaptoethanol) and protease inhibitor (Protease Inhibitor Cocktail, Sigma-Aldrich, St. Louis, MO) with agitation at 4 °C for 11 hr. After two times of centrifugation at 8,000 round per min (rpm) at 4 °C for 5 min each, supernatants were collected and subjected to SDS-PAGE analysis. The SDS-PAGE gels were either stained with Coomassie Blue or transferred to the nitrocellulose membranes (Thermo Scientific) and analyzed by immunoblot analysis. Briefly, the membranes were blocked in 3% milk/phosphate buffer saline (PBS)/0.5% Tween-20 and incubated with primary rabbit anti-EEHV gB antibodies (1:500) in PBS/0.5% Tween-20 for 1 hr at room temperature (RT) with agitation. The membranes were washed three times with PBS, and then incubated with biotinylated goat anti-rabbit IgG secondary antibodies (1:200; EMD Millipore, Billerica, MA) in PBS/0.5% Tween-20 for 45 min at RT. Subsequently, they were washed and incubated with avidin-biotin peroxidase complex (ABC) reagent (Thermo Scientific) for 30 min at RT. Then, the membranes were developed with 3,3′-diaminobenzidine tetrahydrochloride (DAB) substrate and the signals were observed.

### Immunohistochemistry

Immunohistochemistry was performed by employing the ABC method, as described previously^41^. Briefly, the FFPE sections were deparaffinized, rehydrated, and microwaved for 30 min in citrate buffer (pH 6.0). The sections were incubated for 5 min with 3%H_2_O_2_ in methanol, and then blocked for 30 min at RT with PBS containing 5% normal goat serum/1% Tween-20. Thereafter, they were incubated at 37 °C for 2 hr with rabbit anti-EEHV gB antibodies (1:500). After three times of washing with PBS, the sections were incubated for 50 min at RT with biotinylated goat anti-rabbit IgG secondary antibodies (1:200, EMD Millipore), followed by peroxidise coupled ABC (Thermo Scientific). Antibody binding was visualized using DAB for 5 min at RT followed by washing with tap water and counterstaining with Mayer’s hemalum. The negative controls were sections of EEHV1A- and EEHV4-infected calves that were incubated with normal rabbit serum instead of rabbit anti-EEHV gB antibodies, or incubation of non-EEHV-infected tissue sections with rabbit anti-EEHV gB antibodies. The slides were observed and photos were taken under a light microscope.

### Isolation of elephant peripheral blood mononuclear cells (PBMCs)

Isolation of PBMCs from fresh carcass of EEHV4-infected and non-EEHV-infected calves was done, as previously described^36^, with minor modification. Briefly, 20 mL of the blood obtained in the ethylenediamine tetraacetic acid (EDTA)-containing tubes was diluted to an equal volume of PBS. Then, the PBMCs were isolated by density gradient centrifugation on Lymphoprep^TM^ (Alere Technologies AS, Oslo, Norway) at 400 × *g* for 30 min at 4 °C. The interphase cells containing the PBMCs were collected and washed twice with PBS supplemented with 1% fetal bovine serum (FBS; Gibco; Thermo Scientific), and then resuspended in the Roswell Park Memorial Institute (RPMI)-1640 medium (Thermo Scientific) supplemented with 10% FBS, 100 U/mL penicillin G, 100 µg/mL streptomycin, and 0.25 µg/mL amphotericin B. The cells were seeded onto the coverslip-inserted 24-well-microtiter plates (SPL Life Sciences, Gyeonggi-do, Korea) at a concentration of 2 × 10^6^ cells/mL and cultivated at 37 °C with 5% CO_2._ After 2 hr of cultivation, the cells were fixed and immunofluorescent stained, as described below.

### Immunofluorescence

Immunofluorescent staining of elephant PBMCs was done in coverslip-inserted 24-well-microtiter plates, as previously described^42^. Briefly, the cultures were fixed with 4% paraformaldehyde for 15 min at RT, and treated with 0.25% Triton X-100 in PBS (0.25% PBST) for 15 min. Then, the cells were incubated with 1% bovine serum albumin (BSA) in 0.25% PBST for 30 min at RT, followed by incubation with a mixture of primary antibodies diluted with 1% BSA in 0.25% PBST at 4 °C, overnight. The primary antibodies used were rabbit anti-EEHV gB antibodies (1:500) and mouse anti-ionized calcium binding adaptor molecule-1 (Iba-1) antibodies (1:200; EMD Millipore). After three times of washing with PBS, a mixture of secondary antibodies, including FITC–conjugated goat anti-mouse and Cy3–conjugated goat anti-rabbit antibodies (1:200; all from Jackson ImmunoResearch, Suffolk, UK), was incubated for 45 min at RT. The nuclei were counterstained using bisbenzimide (0.01% in ethanol, Sigma Aldrich, St. Louis, MO) for 10 min at RT. The cultures were analyzed and photos were taken under an inverted fluorescent microscope.

### Evaluation of immunolabeling-positive cells

Evaluation of immunofluorescent labeling of PBMCs for specific markers was done using the ImageJ software (National Institutes of Health, Bethesda, MD), and the percentages of positive cells for each marker were calculated, as previously described^42^.

### Statistical analysis

The statistical analyses of immunofluorescent labeling cells were accomplished using GraphPad Prism 5 (GraphPad Inc., La Jolla, CA, USA). The statistical significance was designated as *p* ≤ 0.05.

### Data Availability

All data generated or analyzed during this study are included in this published article, and its Supplementary Information files.

## Electronic supplementary material

Supplementary figure
