# Supplementary material for: Production of antibody against elephant endotheliotropic herpesvirus (EEHV) unveils tissue tropisms and routes of viral transmission in EEHV-infected Asian elephants
Source: Sci Rep. 2018 Mar 16;8:4675. doi: 10.1038/s41598-018-22968-5 (PMC5856810; doi:10.1038/s41598-018-22968-5)
Supplement: Supplementary file 1 — Supplementary figure [file 41598_2018_22968_MOESM1_ESM.doc]

**Production of antibody against elephant endotheliotropic herpesvirus (EEHV) unveils tissue tropisms and routes of viral transmission in EEHV-infected Asian elephants**

Varankpicha Kochagul^1,+^, Saralee Srivorakul^1,+^, Kittikorn Boonsri^1^, **Chalermchart Somgird^2,3^,** Nattawooti Sthitmatee^4^, Chatchote Thitaram^2,3^, Kidsadagon Pringproa^2,4*^

^1^ Veterinary Diagnostic Laboratory, Faculty of Veterinary Medicine, Chiang Mai University, Chiang Mai, Thailand

^2^ Center of Excellence in Elephant and Wildlife Research, Chiang Mai University, Chiang Mai, Thailand

^3^ Department of Companion Animal and Wildlife Clinic, Faculty of Veterinary Medicine, Chiang Mai University, Chiang Mai, Thailand

^4^ Department of Veterinary Biosciences and Veterinary Public Health, Faculty of Veterinary Medicine, Chiang Mai University, Chiang Mai, Thailand

^*^ Corresponding author

E-mail: [kidsadagon.p@cmu.ac.th](mailto:kidsadagon.p@cmu.ac.th) (KP)

^+^ These authors contributed equally to this work.

**Supplementary information**

**S1 Figure.** SDS-PAGE analysis of rabbit sera after immunization with different concentrations of peptide-conjugated KLH carrier protein.

**
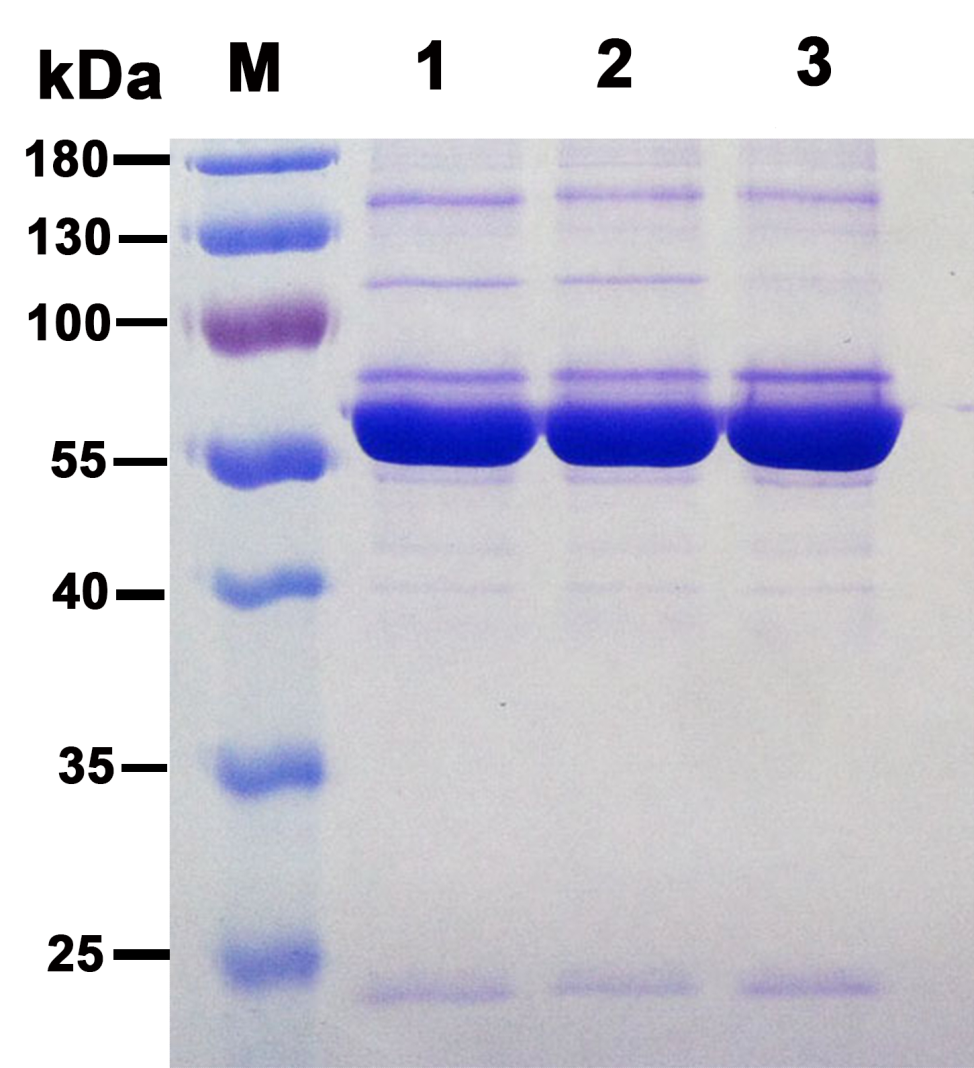
**

Rabbit sera at different concentrations were subjected to SDS-PAGE. Following staining of SDS-PAGE gel with Coomassie Blue, protein sizes of ~25 kDa and ~50 kDa were observed, which indicated the light and the heavy chain of immunoglobulin G (IgG), respectively. Lane M = protein molecular weight marker; lane 1 = rabbit sera immunized with 125 µg/mL; lane 2 = rabbit sera immunized with 250 µg/mL; and lane 3 = rabbit sera immunized with 450 µg/mL.

**S2 Table**. Sequence of EGHV primers used in this study

| **Gene** | | **Sequence (5’-3’)** | **Product size (bp)** | **Acc no.** |
| --- | --- | --- | --- | --- |
| EGHV | Polymerase | Forward: CACCACCTTCGGTTGGTACA | 142 | EU085379.1 |
|  |  | Reverse: CACCTGGTAACTAGGCCAGC |  |  |
|  | Glycoprotein B | Forward :CAAGAGAGGGTGGCCAATGT | 163 | EU085379.1 |
|  |  | Reverse: AGCCAAGGTCGTCCATAAGC |  |  |

Acc.no.: GeneBank accession number
